# Periodontal Plastic Surgery: The Past, the Present, the Future

**DOI:** 10.1111/jre.70151

**Published:** 2026-07-16

**Authors:** Francesco Cairo, Giovan Paolo Pini Prato

**Affiliations:** ^1^ Research Unit in Periodontology and Periodontal Medicine, Department of Experimental and Clinical Medicine University of Florence Florence Italy

## Abstract

Over 40 years of clinical research, Periodontal Plastic Surgery has evolved from a niche topic into an issue of great collective interest in Periodontology.

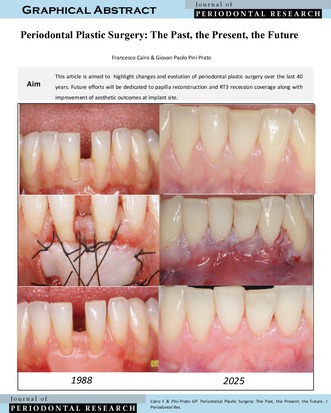

## 1 Introduction

By long‐established habit, people have always spoken of Past, Present, and Future in art, in social activities, and particularly in medical sciences. Changes can be rapid, tumultuous, sometimes controversial, and foreshadow the development of new knowledge in a more or less near future.

Although this model of thought (past–present–future) is intuitive and easy to understand, when examined carefully and critically, it does not allow us to define with certainty the temporal boundary between past, present, and future.

On March 21, 1955, in a letter to the recently deceased friend M.A. Besso, Albert Einstein wrote:

For those of us who believe in physics, the distinction between past, present and future is only a stubbornly persistent illusion.

Thus, encouraged by the thought of the great Einstein, even in the history and evolution of periodontal plastic surgery it is impossible to determine with certainty the boundary between past, present, and future, as variables such as knowledge, techniques, materials, technologies, practitioners, and even patients change rapidly and progressively over time.

A possible interpretation of changes in Periodontal Plastic Surgery that have occurred over time (past), current approaches (present), and the projections for a not‐so‐distant future (future) can be made by evaluating three fundamental models: surgical techniques, objectives, and the methodology of scientific research.

Therefore, the purpose of this article is to present changes and evolution of Periodontal Plastic Surgery using the past–present–future temporal trend highlighting connections and overlaps among different eras.

## 2 The Past

Classical paradigms postulated that a large amount of attached keratinized tissue (KT) was a key factor for maintaining periodontal health and preventing periodontal infection. Descriptive studies by Bohannan [1, 2] showed that after “*denudation*” or “*partial‐thickness flap technique*” the wound area was filled with granulation tissue from periodontal ligament leading to increased KT after bone crest resorption. Healing following these procedures often resulted in increased gingival recession due to extensive bone loss, even if some KT increase was apically detectable. These techniques were progressively neglected when the use of free gingival grafting (FGG) became universally accepted. Evidence from large studies demonstrated FGG is the most effective technique for increasing KT in height and thickness and the achieved outcomes are stable over time [3]. The clinical success and wide application of FGG determined the golden era of the *Mucogingival Surgery*, defined as “Surgical procedures designed to preserve gingiva, remove aberrant frenum, or muscle attachment and increase the depth of vestibulum” [4].

**FIGURE 1:**
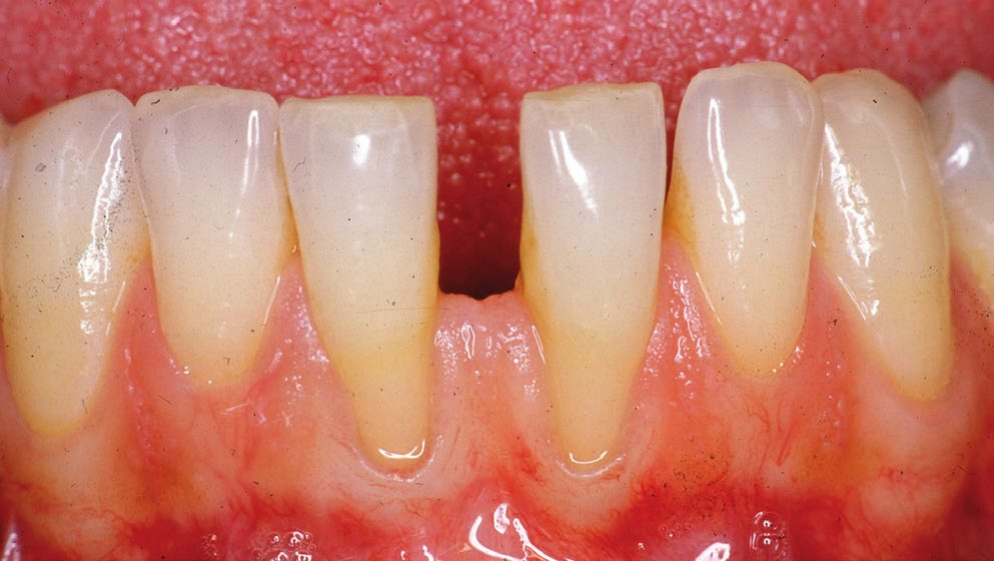
RT 2 recessions at lower incisors. The case was treated with a free gingival graft in 1988.

**FIGURE 2:**
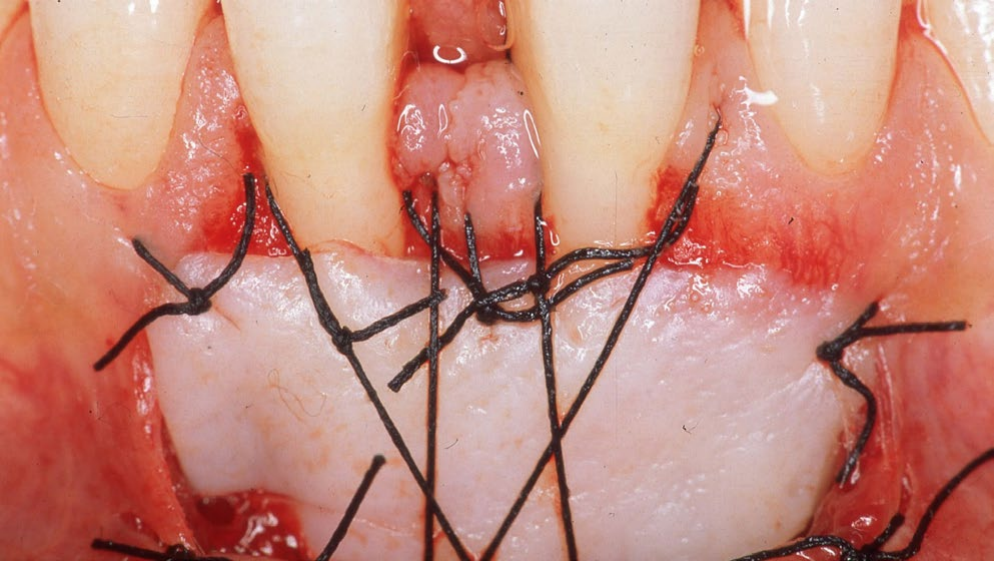
The free gingival graft was sutured.

**FIGURE 3:**
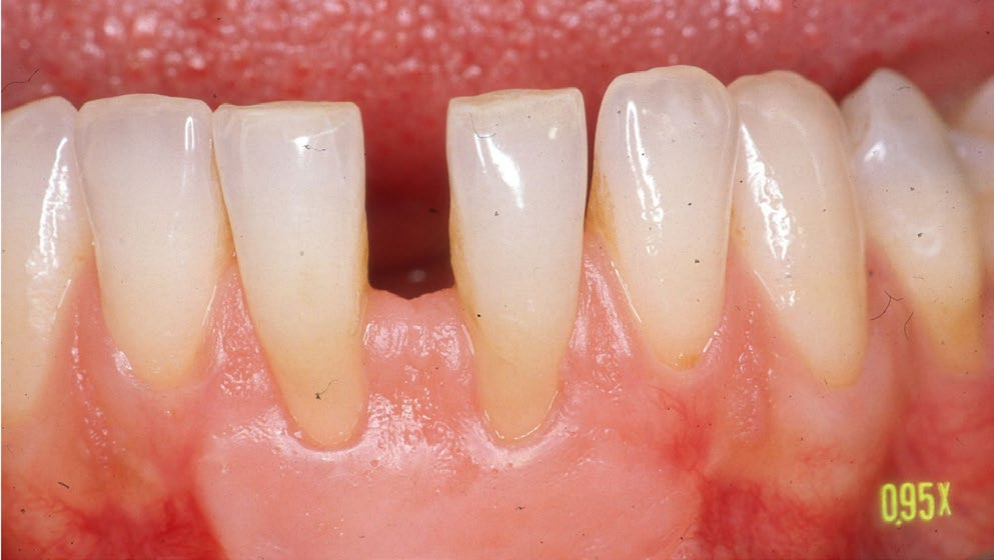
Healing after 6 months with partial root coverage.

In 70's, the FGG use was standardized also for root coverage procedure. In fact, a modification of this technique (two step procedure) was firstly proposed in 1975 by Bernimoulin et al. [5]. Two months after graft transplantation, a coronally advanced flap with two vertical incisions was applied to obtain root coverage. Furthermore, based on his classification into 4 gingival recessions classes, Miller [6] suggested an attempt of root coverage prognosis, describing full root coverage possible for Class I and II (intact interdental papillae/bone), partial coverage predictable for Class III (partial loss of interdental bone/papilla) and no root coverage attainable for Class IV. This early attempt of outcome prediction was mostly focused on FGG application, since integrity of interdental papilla are critical to support graft healing (Figures 1, 2, 3).

### 2.1 Connective Tissue Grafts

In the 1980s, a revolutionary idea arose that stimulated a great improvement in the surgical technique of root coverage: the subepithelial connective tissue graft (CTG) proposed by Langer and Langer [7]. Based on conservation of tissue specificity after transplantation of gingival and alveolar mucosa [8], the graft was placed under a split‐thickness flap that was retained to partially cover the CTG, as opposed to being entirely left exposed as in the FGG procedure. As a result, the autogenous soft tissue graft received a double nutritional supply from both overlying flap connective tissue and underlying periosteal bed, thus supporting an improvement in root coverage outcomes compared with FGG [7].

During the same year, Raetzke [9] modified the CTG technique, placing the graft beneath an envelope flap, avoiding papillae incision and laying the groundwork for modern tunneling techniques. A few years later, Nelson [10] proposed a modification of the CTG approach, describing it as a “bilaminar grafting” technique composed of two biological layers: a free connective tissue graft and an overlying pedicle flap. Evaluating the outcomes of this procedure, Nelson stated, “bilaminar grafting techniques have the potential to be employed in the future” [10].

Finally, Allen and Miller [11] presented a simplified technique to correct shallow recessions with favorable tissue phenotype (presence of at least 3 mm of apical KT) by utilizing a “coronally advanced” flap (CAF) of the existing gingiva, incorporating triangular surgical papillae, without the need for supplementary autogenous soft tissue grafts. This approach, although limited in a similar fashion to the lateral pedicle flap, provided the basic feature of the later and the most current root coverage procedures.

### 2.2 Guided Tissue Regeneration (GTR)

In the early 90's, based on the principle of guided tissue regeneration (GTR), several techniques were developed to treat gingival recessions [12]. Even if the results of this approach were surprising particularly due to the formation of new attachment on the denuded root surfaces, the operative difficulty of surgery as well as the magnitude of clinical benefits, economic cost, and high prevalence of complications following this sophisticated technique caused its unemployment in modern Periodontal Plastic Surgery [13].

### 2.3 Tunnel Technique and Multiple Coronally Advanced Flap

The use of a CTG underneath an envelope that preserved the papillae represented an early attempt at tunneling aimed at maximizing host healing and revascularization, while still achieving root coverage for single and multiple gingival recessions, with improved esthetics. Initial macro‐surgical techniques [9, 14, 15] established the biological rationale, indications, and clinical applications of tunneling techniques.

In the same years, Zucchelli and de Sanctis [16] modified the original coronally advanced flap introduced by Bernimoulin et al. [5] by applying a multiple coronally advanced envelope flap (MCAF); the vertical releasing incisions were eliminated to improve vascular network thus supporting flap healing and esthetic outcomes. MCAF was traditionally introduced to treat multiple recessions in the incisor‐first molar region.

This new trend in moving behind the pure improvement of gingival quantity toward excellent soft tissue integration supported the introduction of *Periodontal Plastic Surgery* as “Surgical procedures performed to prevent or correct anatomical, developmental, traumatic or plaque induced defects of gingiva, alveolar mucosa, and bone” [17]. These new concepts were progressively applied also for ridge defect treatment [18]. Moreover, the introduction of tunneling procedure and multiple coronally advanced flap has allowed clinicians of the new millennium to evaluate modern treatments beyond the coverage of single recession.

## 3 The Present

From the beginning of the new millennium, evidence‐based dentistry has revolutionized Periodontology. The number of randomized controlled clinical trials (RCTs) rapidly increased, showing that coronally advanced flap design is the most supported procedure in Periodontal Plastic Surgery. Interestingly, “predictors” of root coverage were focused on by subsequent studies demonstrating that flap thickness [19], tension [20], position at the time of suture [21] and baseline recession depth [22] were the most relevant surgical‐related factors for CAF technique. Conversely, add of CTG was improved in shape and position to obtain optimal esthetic outcomes [23]. Basing on this sound evidence, the combination CAF + CTG is today considered the gold standard technique for single recession treatment, especially in cases of deep recession associated with thin phenotype [13, 24]. A new classification system based on the loss of interdental attachment (iCAL) was accepted to predict clinical outcomes [25].

Furthermore, in case of recessions associated with non‐carious cervical lesions (NCCL), a combined restorative and periodontal treatment is nowadays often used since approximately half of treated recessions may show partial or complete CEJ abrasion [26]. Sophisticated restorative techniques require careful reconstruction limited to the CEJ area, avoiding involvement of the exposed root area where pure soft tissue reconstruction is accomplished [27, 28, 29]. Similarly, a high level of evidence is available in the scenario of multiple recession treatment. The envelope coronally advanced flap has proven its efficacy alone or in combination with CTG [30, 31].

**FIGURE 4:**
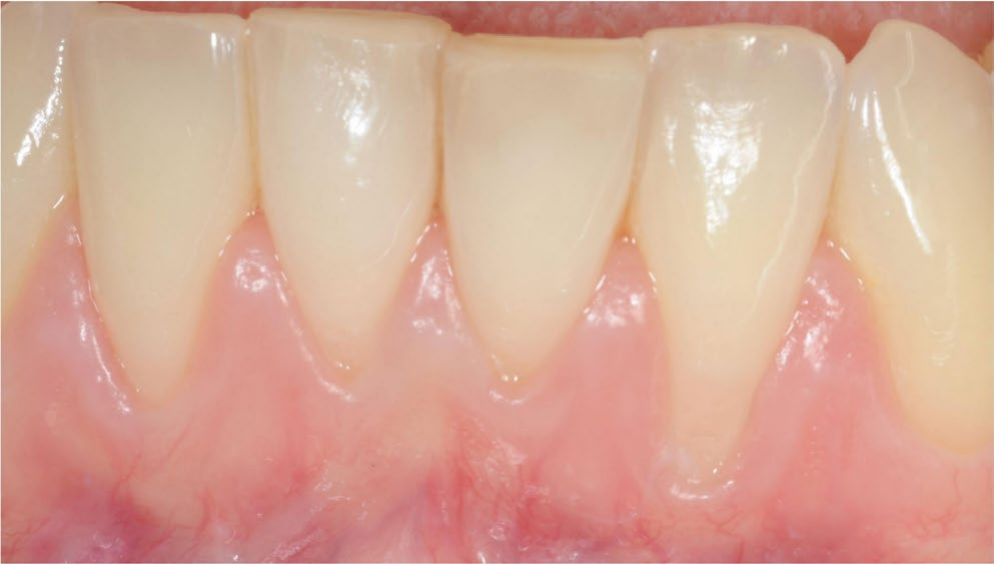
Baseline recessions at lower incisor. The case was treated with a coronally advanced flap with tunnel and connective tissue graft (year 2025).

**FIGURE 5:**
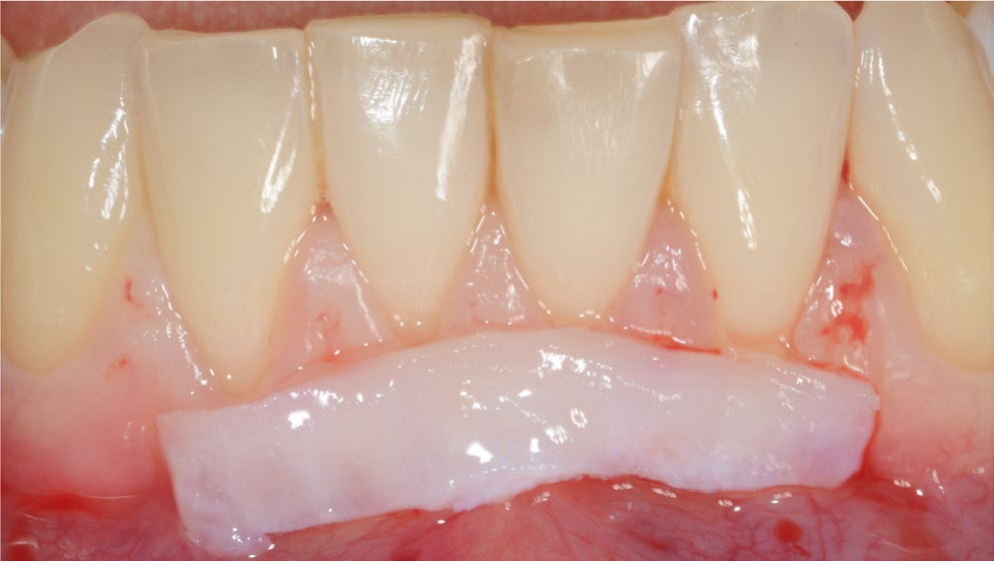
The connective tissue graft was harvested.

**FIGURE 6:**
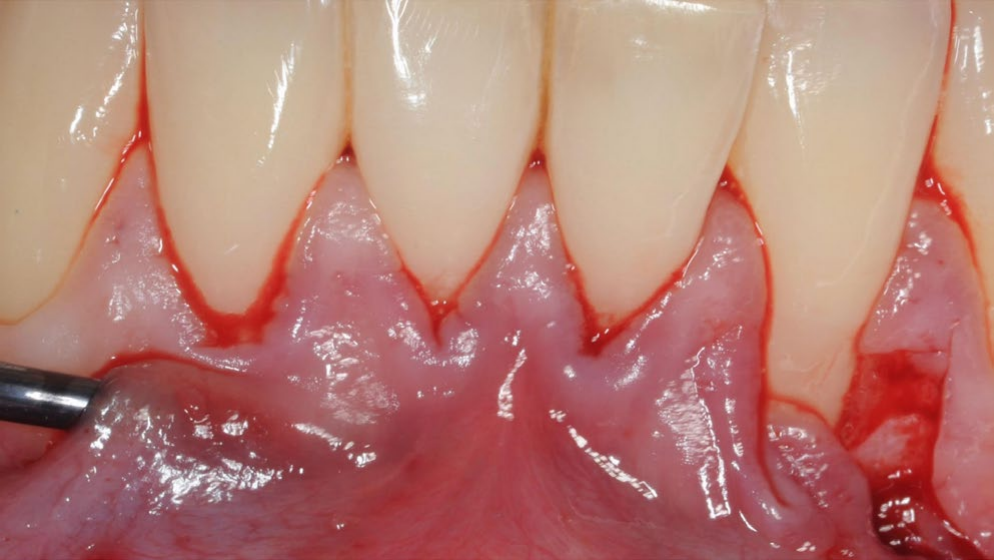
Different approaches were mixed, including a coronally advanced flap, a tunnel technique, and a lateral vertical incision (vista incision).

**FIGURE 7:**
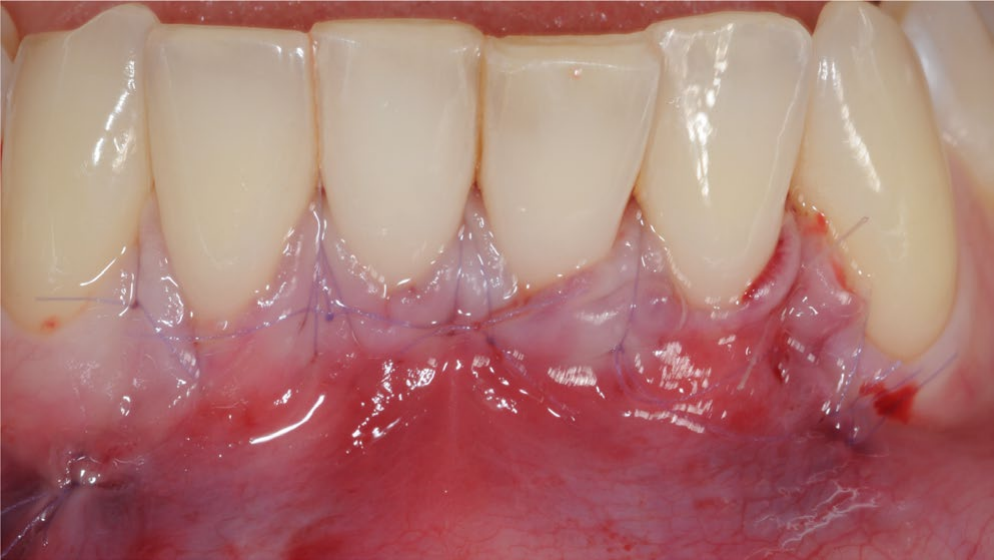
After connective tissue graft insertion, the flap and tunnel were coronally advanced.

**FIGURE 8:**
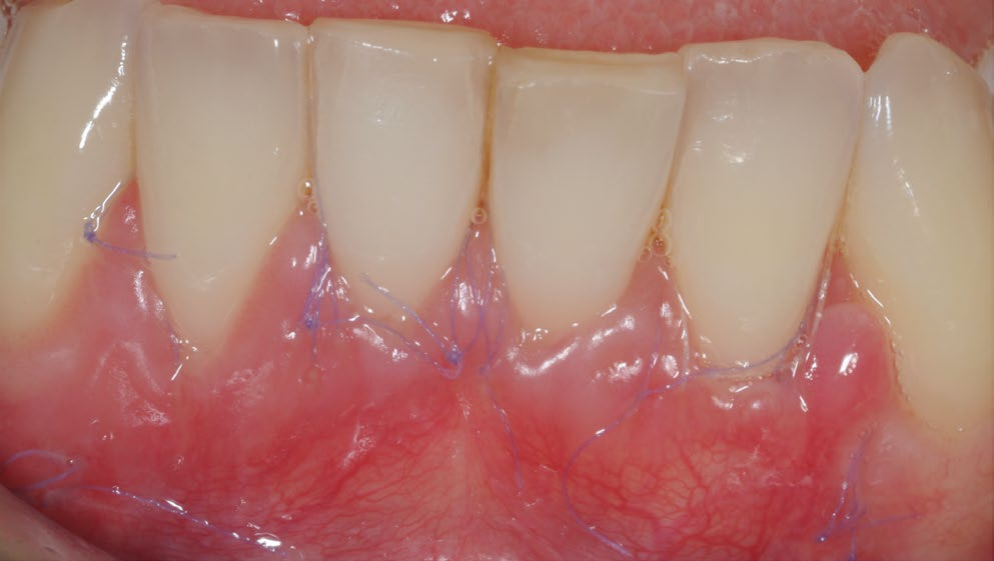
Early healing after 10 days.

**FIGURE 9:**
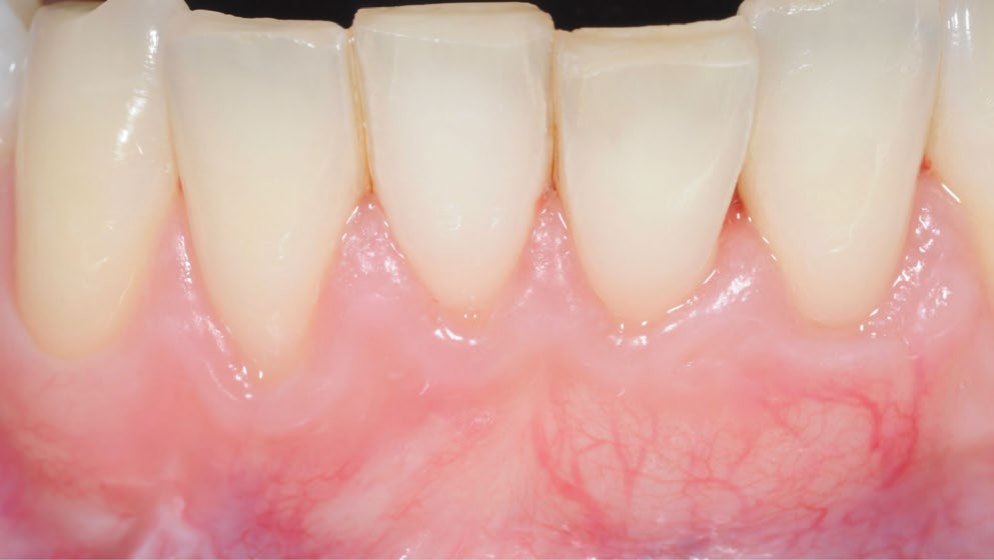
Soft tissue healing and root coverage after 6 months.

Advances in the understanding of oral soft tissue biology led to the adoption of improved armamentarium for tunneling procedures, with microsurgical blades and tunneling knives. Subsequent studies further developed variations of tunneling approaches by applying microsurgical blades and specifically tailored knives, including supra‐periosteal versus subperiosteal tunneling, tunnel‐specific suturing techniques for graft stabilization, coronal positioning of the advanced tunnel and laterally recession closure [32, 33, 34], thereby creating the various branches of tunneling procedures that are now widely adopted in clinical practice (Figures 4, 5, 6, 7, 8, 9).

Available evidence seems to support the use of biomaterials, mainly collagen matrix or enamel matrix derivatives, as able to improve the outcomes of CAF at both single and multiple recessions [24, 25, 26, 27, 28, 29, 30, 31, 32, 33, 34, 35]. However, if compared to CTG, the use of biomaterials offers better postoperative morbidity but a lesser amount of root coverage at short and long‐term observations [36].

Along with the predictability of clinical outcomes, two important issues are currently critical in modern periodontal plastic surgery: professional esthetic outcomes evaluation and patient‐reported experiences (PREs) and outcomes (PROs). Professional esthetic outcomes evaluation is critical to evaluate the outcomes of root coverage, since the amount of recession reduction is an effective parameter in terms of quantity but not able to capture outcome quality. Some indices are commonly used, including the Root coverage esthetic score (RES) [37, 38] that merges quantity of root coverage with quality assessment of gingival color, scar tissue presence, alignment of MGJ and final characteristics of gingival margin, thus helping clinicians to critically assess final healing. Recent research has proven that the use of CAF with CTG has the highest impact on final RES values [39].

On the other hand, the concept of patient evaluation and experience has become a critical issue in clinical trials too [40]. These parameters such as Visual Analogue Scale (VAS) and Oral Health Impact Profile (OHIP‐14) are widely used to evaluate the impact of procedure on healthy people, particularly to capture data on patient morbidity and occurrence of postsurgical or esthetic complications as well as to support clinicians evaluating the cost–benefit ratio of surgery [40].

Notably, current concept of root coverage prognosis has changed compared to Miller's paradigms. Data from RTCs proven that it is possible to completely cover gingival recession with some loss interdental clinical attachment (RT2) mainly using CTG at both single [41] and multiple recession defects [42]. These data are corroborated by several biological insights regarding the healing process of bilaminar procedures, including a very fast revascularization process [43].

### 3.1 Peri‐Implant Plastic Surgery

Periodontal Plastic Surgery has progressively gained also interest in the scenario of implant dentistry. In fact, esthetic failures deriving from the insurgence of peri‐implant soft tissue dehiscence/deficiencies are a common finding after implant therapy [44]. Although initial data suggested that peri‐implant soft tissue dehiscence was considered a natural occurrence of the implant therapy [45], in the new millennium emerging evidence suggested the importance of soft tissue augmentation at implant site for the maintenance of tissue height/level, patient comfort, and esthetics [46]. Clinicians faced similar challenges but were facilitated by the use of well‐tested techniques and materials than those found by periodontists 70 years ago treating mucogingival deformities at natural teeth.

Later, Burkhardt et al. [47] first applied CAF with CTG for the treatment of peri‐implant soft tissue dehiscence, setting the baseline for further research in the field of peri‐implant soft tissue therapy. Improved surgical techniques were then proposed by using only soft tissue augmentation with CTG [48] or combining the CAF + CTG procedure with modifications of the prosthetic components to improve predictability of treatment [49]. Modern longitudinal studies suggested that soft tissue augmentation is critical to prevent the risk of recession in the long term, and success of implants in the esthetic area is associated with proper combination of guided bone regeneration and CTG [50].

Active research is currently focused on achieving a better understanding of the relative contributions of hard tissue and soft tissue phenotypes to the final outcomes of implant therapy, including long term survival, esthetics, and patient satisfaction. Recent consensus reports have emphasized that soft tissue phenotype augmentation reduces the risk of peri‐implant soft tissue dehiscence and improves esthetics, patient comfort during oral hygiene procedures, and long‐term stability compared with non‐augmented sites [51].

Dental implants offer the unique opportunity to remove the prosthesis and benefit from spontaneous soft tissue thickening and wound closure. In addition, modification of the prosthetic supra‐structure through the use of narrower abutments may allow selective tissue thickening in critical areas. However, these prosthetic advantages of dental implants over natural dentition are counterbalanced by biological disadvantages, including the reduced vascularity of peri‐implant tissues compared with periodontal counterparts [47, 48, 49, 50, 51].

## 4 The Future

In term of number of published RTCs, Periodontal Plastic Surgery is one of the most supported procedures in Dentistry. This huge level of evidence will offer clinicians a large range of options of treatments in respect to the type of defect, locations, and clinical‐related parameters. It is rational to hypothesize that novel biomaterials will be available in order to reduce morbidity of the procedures, even if the CTG will always be considered the gold standard. Hybridized techniques connecting CAF and tunnel procedures will probably be very useful in the future along with a site‐specific use of CTG. A large effort will probably be dedicated to the treatment of RT3 recession and associated loss of interdental papilla in order to improve esthetic outcomes also in periodontal patients. Finally, an esthetic prognosis will be developed to forecast not only clinical but also esthetic outcomes of the procedures.

Periodontal plastic surgery continues to advance into the future with equal emphasis on diagnostics and therapeutics. Mention should be given to the use of noninvasive diagnostics currently under investigation which open the doors for better understanding of tissue biology as well as improved communication and personalized medicine approaches, such as intraoral ultrasonography to assess vascular perfusion [52], or digital photography for the application of telemedicine in periodontal plastic surgery [53].

## 5 Conclusions

Looking back over 40 years of clinical research, Periodontal Plastic Surgery has evolved from a niche topic for a few periodontists into an issue of great collective interest in Periodontology. Restoring lost soft tissue at exposed roots has become a reliable procedure in terms of clinical and esthetic outcomes. In this perspective, techniques developed around teeth are successfully transferred to dental implants, contributing to excellent results of modern implantology. The quality of clinical research and the learning curve of the operator will always be critical in the achievement of outcomes for these very sophisticated surgeries.

## Conflicts of Interest

The authors declare no conflicts of interest.

## Data Availability

The data that support the findings of this study are available from the corresponding author upon reasonable request.
